# Decolonizing global health: an agenda for research

**DOI:** 10.1186/s12913-023-09834-5

**Published:** 2023-08-07

**Authors:** Henry Zakumumpa, Nafissatou Diop, Robert Kaba Alhassan

**Affiliations:** 1 Makerere University, Kampala, Uganda; 2 Canadian Association for Global Health, Ottawa, Canada; 3 Health Systems Global, Ottawa, Canada; 4 University of Health and Allied Sciences, Volta Region, Ghana

## Abstract

This editorial outlines the background to the BMC Health Services Research collection on decolonizing global health. The discourse on decolonizing global health is gaining increasing momentum. These persistent ‘voices’ have coalesced into a ‘movement’. Several commentators have critiqued the way global health continues to be structured and practiced. The colonial roots of global health dominance have come under an unprecedentedly intense spotlight amid pressure for reform.

## Main body

In this editorial we set the context and invite contributions to the *BMC Health Services Research* collection on decolonizing global health. In this connection, we wish to highlight some issues on the current global health landscape.

The colonial legacies of global health educational institutions, the content of their curricula, their training pipelines, and the tendency to privilege knowledge production from these training institutions has engendered debate [1]. Other emerging global health issues focus on how Low and middle income countries (LMICs) should avoid falling into another round of colonial capture through emerging global health research agendas (e.g. climate change, planetary health, One Health). However, more knowledge is warranted in unpacking and methodically documenting these notions.

It has been observed that transnational global health ‘partnerships’ are steeped in power asymmetries [2]. Some critics have noted that researchers based in High-Income Countries (HIC) frequently frame the research question(s), lead the grant writing and ‘host’ research grants while partners based in LMICs have limited, and often peripheral roles, such as data collectors or ‘native informers’. Mamdani refers to the latter as ‘researchers who are used to provide raw material – in form of data – to foreign academics who process it and then re-export it back to Africa’ [3]. The practice of parachute research to further the career of scientists from HICs with little input or benefit for LMIC-based scientists and to the population health of countries where the research was conducted has come under critical spotlight [4]. The politics of global health research funding is worthy of critical reflection under this special collection [5]. A related thorny issue is the debate on inequities in authorship on peer-reviewed articles, particularly when publishing in the so-called prestigious journals, most of which are HIC-based [4].

Beyond individual researchers, the power imbalance and differentials in global health institutions depending on where they are located has also come into critical focus. Organizations in LMICs are by no means themselves devoid of internal power hierarchies and this deserves attention under this collection. Another overlooked dimension is the identity and legitimacy dilemmas experienced by global health researchers who are from the LMIC-diaspora but are based in HIC. They are sometimes perceived as “transplants” by whichever side they are dealing with. The positionality of contributors on the subject of decoloniality deserves reflexivity in the kind of submissions we are calling for. What ‘baggage’ do commentators bring to this discourse based on country (colonial) histories, the power that is wielded in existing professional networks, *alma mater* complexes or the notion of race and privilege [6–8].

Another debate that has arisen is around the political correctness of expressions such as “technical assistance” and “capacity building”-the unpalatable connotation that there is no local knowledge and competencies in LMICs. A clear message of the decolonizing global health movement is that learning is a two-way street such as instances entailing ‘reverse innovation’ where innovations first developed in LMICs have found their way in HICs. Terminologies must convey the ethos of equity in partnerships.

Although global health inequities have been well described, the underpinning contexts that give rise to these imbalances have not been sufficiently understood. For instance, we invite political economy analyses that attempt to unearth the complexities underlying global health inequities or factors that perpetrate or perpetuate them. Novel insights on this topical debate in decoloniality in global health will be considered.

The reawakening of a consciousness for the need for equity and the quest for a ‘fairer’ global health order is an unrelenting wave [5]. How can colonial vestiges be dismantled in ways that promote ‘local ownership’ and improve population health outcomes [9]? Although the concept of ‘multipolarity’ has been introduced as one of the pathways for achieving more equitable power sharing and agenda setting in calls for decolonization [10], this concept is still not well developed. We welcome papers that explore this concept further.

In this collection we welcome thought-provoking contributions that rise above ‘the bandwagon effect’ and ones that aim to move beyond rhetoric to action. We invite high-quality case-studies, retrospective analyses, secondary analyses of data, theory-informed approaches, and systematic reviews as well as articles that seek to advance current knowledge or map a path forward.

## Data Availability

Not applicable.
